# Finishing and polishing systems influence the roughness and color stability of acrylic and bis-acryl composite resins

**DOI:** 10.4317/jced.61598

**Published:** 2024-06-01

**Authors:** Mauro-Gustavo-Amaral Brito, Marlus-da Silva Pedrosa, Ariel-José Bona, José-Augusto Rodrigues, Flávia-Lucisano-Botelho do Amaral, Daiane-Cristina Peruzzo, Fabiana-Mantovani-Gomes França

**Affiliations:** 1 Christus Faculdade do Piauí, Piripiri, Piauí, Brazil; 2 São Leopoldo Mandic Institute and Dental Research Center, Campinas, SP, Brazil; 3 The University of North Carolina at Chapel Hill, Chapel Hill, NC, United States of America

## Abstract

**Background:**

To evaluate the effect of different finishing and polishing systems on the surface roughness and color changes of bis-acryl (Protemp 4 - 3M ESPE- St. Paul, USA; Structur 3 - Voco, Cuxhaven, Germany) and chemically activated acrylic materials (Duralay - Reliance, SP, Brazil).

**Material and Methods:**

Specimens (10 x 2 mm) thick were prepared for each material. The specimens were subjected to polishing and finishing procedures with aluminum oxide discs (Diamond Master - FGM, Joinville, Santa Catarina, Brazil) and spiral rubber disks (Sof-Lex - 3M ESPE, Germany). The control did not receive any polishing and finishing procedures. Surface roughness and color measurement values were obtained after the finishing and polishing procedures and immediately after 30 days of storage in water, coffee, and red wine. Data for each material were analyzed by One-Way ANOVA (*p*<0.05).

**Results:**

The polishing with aluminum oxide discs was able to affect the initial surface roughness values of chemically activated acrylic material (*p*<0.05). After immersion in staining solutions, lower ∆E values were only observed for the bis-acryl composite resins compared to the control group (*p*<0.05).

**Conclusions:**

The finishing and polishing systems influenced the surface roughness and color stability of the materials tested. The chemically activated acrylic resin showed lower surface roughness and higher color stability than the bis-acryl materials.

** Key words:**Acrylic resin, bis-acryl resin, provisional restoration.

## Introduction

There are two primary categories of resins commonly used in dentistry for temporary restorations: chemically activated acrylic resins (CAARs) and bis-acryl composite resins (1-3). CAARs are formulated with the monomer methyl methacrylate, whereas bis acryl composite resins incorporate bifunctional acrylates in their composition. These bifunctional acrylates facilitate cross-linking and are combined with inorganic fillers (1,3-5).

CAARs and bis-acryl composite resins are recommended for creating provisional indirect restorations such as inlays, onlays, crowns, veneers, and bridges (1,6). Additionally, CAARs are used in the fabrication of dentures, dental splints, orthodontic appliances, and various other applications. CAARs are the most utilized material in dentistry for fabricating provisional restorations and dentures due to their cost-effectiveness and satisfactory mechanical properties (6).

When considering provisional restorations, obtaining adequate anatomy and a smooth surface of the material by using finishing and polishing systems is an important factor in reducing biofilm accumulation and, consequently, caries and periodontal diseases (7,8). In restorative dentistry, finishing is defined as a procedure to shape the overall structure of restoration to achieve the desired anatomy, while polishing is the process of smoothing out roughness and eliminating scratches caused by finishing instruments (8,9).

Despite the use of finishing and polishing, methacrylate-based materials used for temporary restorations may still undergo changes in roughness and color changes due to incomplete polymerization, water and pigment absorption, chemical reactivity, diet and medications, and overall oral hygiene (10). Moreover, the effect of polishing systems on the mechanical and optical properties of CAARs and bis-acryl composite resins has been reported to be material-dependent (11).

This study aimed to evaluate the effect of different finishing and polishing systems on the surface roughness and color changes of bis-acryl (Protemp 4 - 3M ESPE- St. Paul, USA; Structur 3 - Voco, Cuxhaven, Germany) and chemically activated acrylic (Duralay – Cotia, Brazil) materials. The null hypotheses tested were: 1 – roughness would not be influenced by finishing and polishing procedures; 2 – the finishing and polishing procedures would not have a significant effect on the color changes of the tested materials.

## Material and Methods

-Specimen Preparation

This in vitro experimental protocol was approved by the Ethics Committee of the School of Dentistry of the São Leopoldo Mandic Institute and Dental Research Center (Protocol# 2019/0189). One CAAR (Duralay - Reliance, SP, Brazil) and two bis acryl (Protemp 4 - 3M ESPE- St. Paul, USA; Structur 3 - Voco, Cuxhaven, Germany) resins were evaluated ( Table 1). Specimens of 10 mm in diameter and 2 mm thick were obtained (n = 10 / group). The sample size was calculated according to previous studies (1,12). The CAAR was handled according to the manufacturers’ instructions. Briefly, the powder was added to the liquid in a glass recipient, and the complete imbibition of the powder was carried out with the aid of a #24 spatula. For the bis acryl resin specimens, the content was dispensed in a single increment directly into the matrix with the aid of mixing tips. A 1-mm-thick glass slide was positioned over the matrix with a 1-kg weight for 30 s to ensure the complete filling of the material inside the matrix and the smoothing of the surface (1,12).

-Finishing and polishing procedures

**Figure 1:**
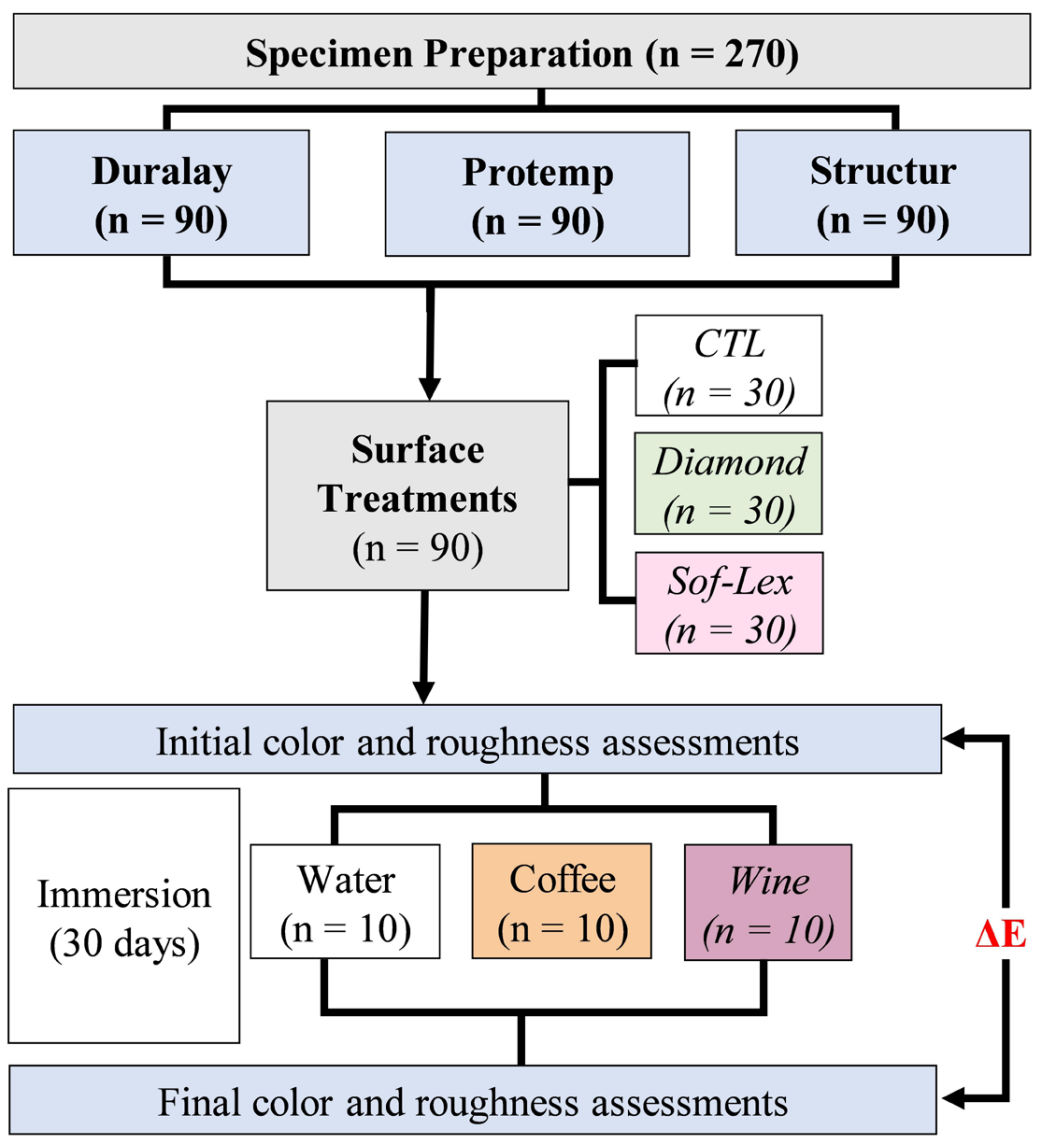
Flowchart of the experimental design.

After complete polymerization, specimens were subjected to polishing and finishing procedures according to the following groups (n = 10 / group): control (without any polishing and finishing procedure), polishing with aluminum oxide discs (Diamond Master - FGM, Joinville, Santa Catarina, Brazil) and spiral rubber disks (Sof-Lex - 3M ESPE, Carl-Schurz-Str, Neuss, Germany) ( Table 2). The complete flowchart of the experimental design is presented in Figure 1.

To standardize the finishing and polishing procedures protocol, the process was performed by the same operator with instruments attached to a handpiece at 20.000 rpm, for 40 s, using mild hand pressure and under constant water irrigation. After checking the specimen’s final dimensions using a digital caliper (Lukas Tools Digital Caliper 300 mm, Vogel, Kevelaer, Germany), specimens were cleaned in an ultrasonic vat (13). Then, the samples (n = 10 / group) were stored in deionized water, red wine (11% vol. alcohol - Saint Germain® Assemblag, Brazil), and coffee (Maratá Traditional, Brazil) and kept in lightproof containers at 37ºC for color measurements. The solutions were renewed every two days for 30 days. The specimens were cleaned in running water for 30 s per face and immersed in distilled water in an ultrasonic vat for 6 min before assessments (1,12).

-Surface roughness

The surface roughness of the specimens was measured after the finishing and polishing procedures and immediately after 30 days of storage in staining solution by using profilometer (TR200, Beijing TIME High Technology, Beijing, China). For each specimen, three measurements were performed in three directions (vertical, horizontal, and oblique) using the diamond stylus with a 5 μm radius, at a 0.25 mm cut-off value, with a 1.25 mm total length and Gaussian filter. The mean value of all peaks and valleys (Ra) and the maximum distance between the highest peak and the deepest valley (Rz) in the measured profile were recorded. For each specimen, the average value from three measurements was calculated (13,14).

-Color Measurements

**Figure 2:**
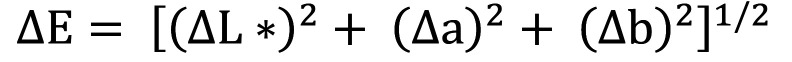
Formula.

A spectrophotometer (VITA Easy shade® - VITA) was used for all color measurements. Measurements were obtained after finishing and polishing procedures and after 30 days of storage in staining solutions. Black background, operator, place, and lighting conditions were standardized for all samples. The samples were dried with gentle air pressure before the 30-day color measurements. All color measurements were presented according to the Commission Internationale de l’éclairage L*, a*, and b* values. The CIELAB system provides values for L*a*b*, where L* represents lightness, a* the red-green axis, and b* the yellow-blue axis. Color differences (ΔE) were calculated using the following formula (15), (Fig. 2):

-Statistical analysis

Data were analyzed using GraphPad Prism Software 7.0 (GraphPad Software, San Diego, CA, USA) with a significance level of 5%. Normal data distribution and homogeneity of variances were verified by Shapiro-Wilk and Levene tests, respectively. One-way ANOVA with the Tukey test was used to analyze the differences in color changes and roughness between materials.

## Results

-Roughness

**Figure 3:**
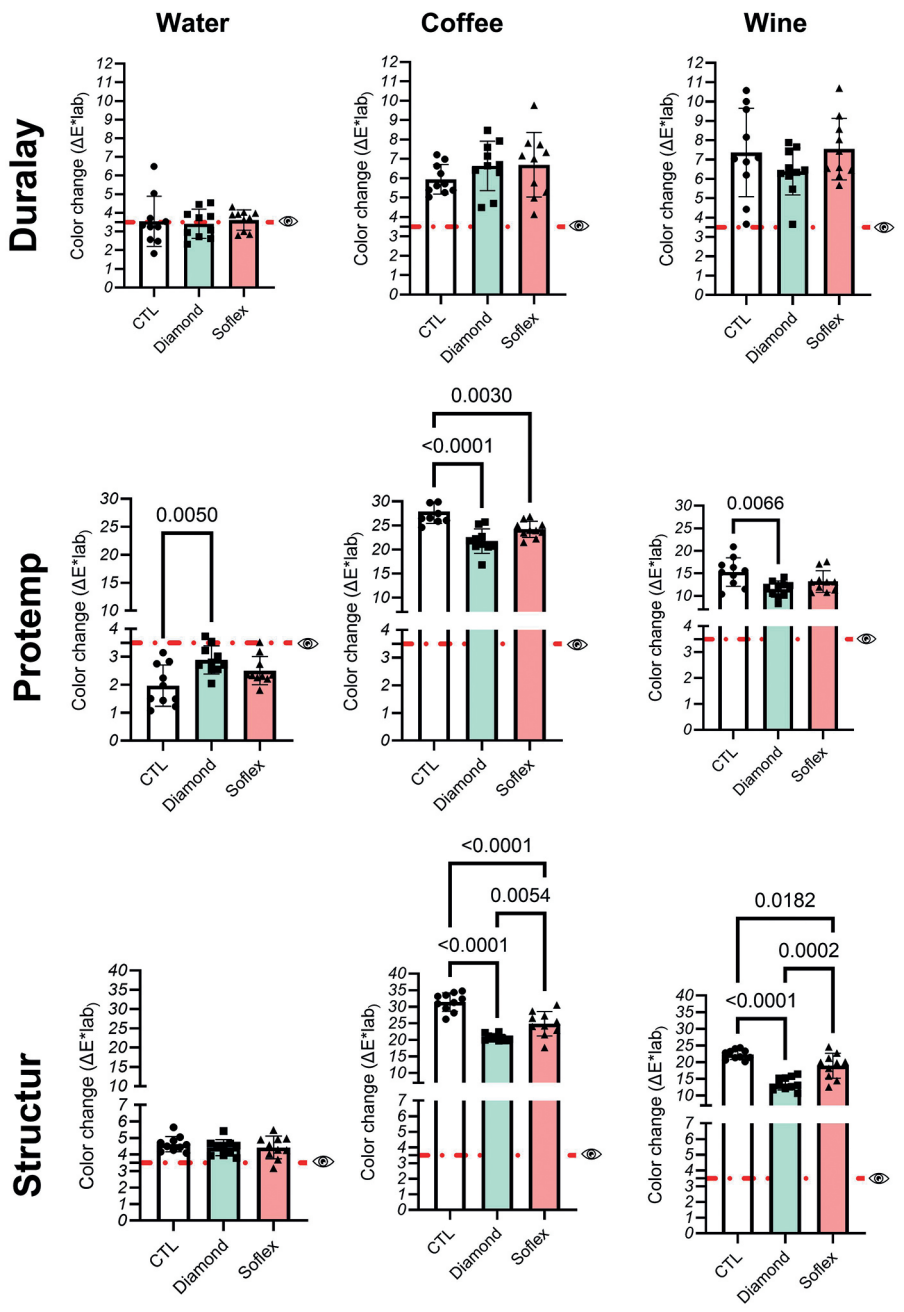
Mean and standard deviation in color changes ΔE values of the materials. p-values are presented when a significant difference is observed between groups by one-way ANOVA followed by Tukey’s tests (*p*< 0.05, n = 10/group). The red dashed line indicates the threshold of perceptibility.

Figure 3 shows that the polishing with aluminum oxide discs (Diamond group) was able to affect the initial surface roughness of Duralay and Protemp (*p* < 0.05). Interestingly, the surface roughness values decreased for Duralay and increased for Protemp (*p* < 0.05). When considering the immersion in staining solutions, the Diamond polishing system was able to significantly decrease the surface roughness values of the acrylic resin (Duralay) when immersed in coffee and wine (*p* < 0.05). For the bis-acryl composite resin Structur, no changes in surface roughness were observed (*p* < 0.05) after the finishing and polishing procedures and immersion in the staining solutions (*p* > 0.05).

-Color changes

**Figure 4:**
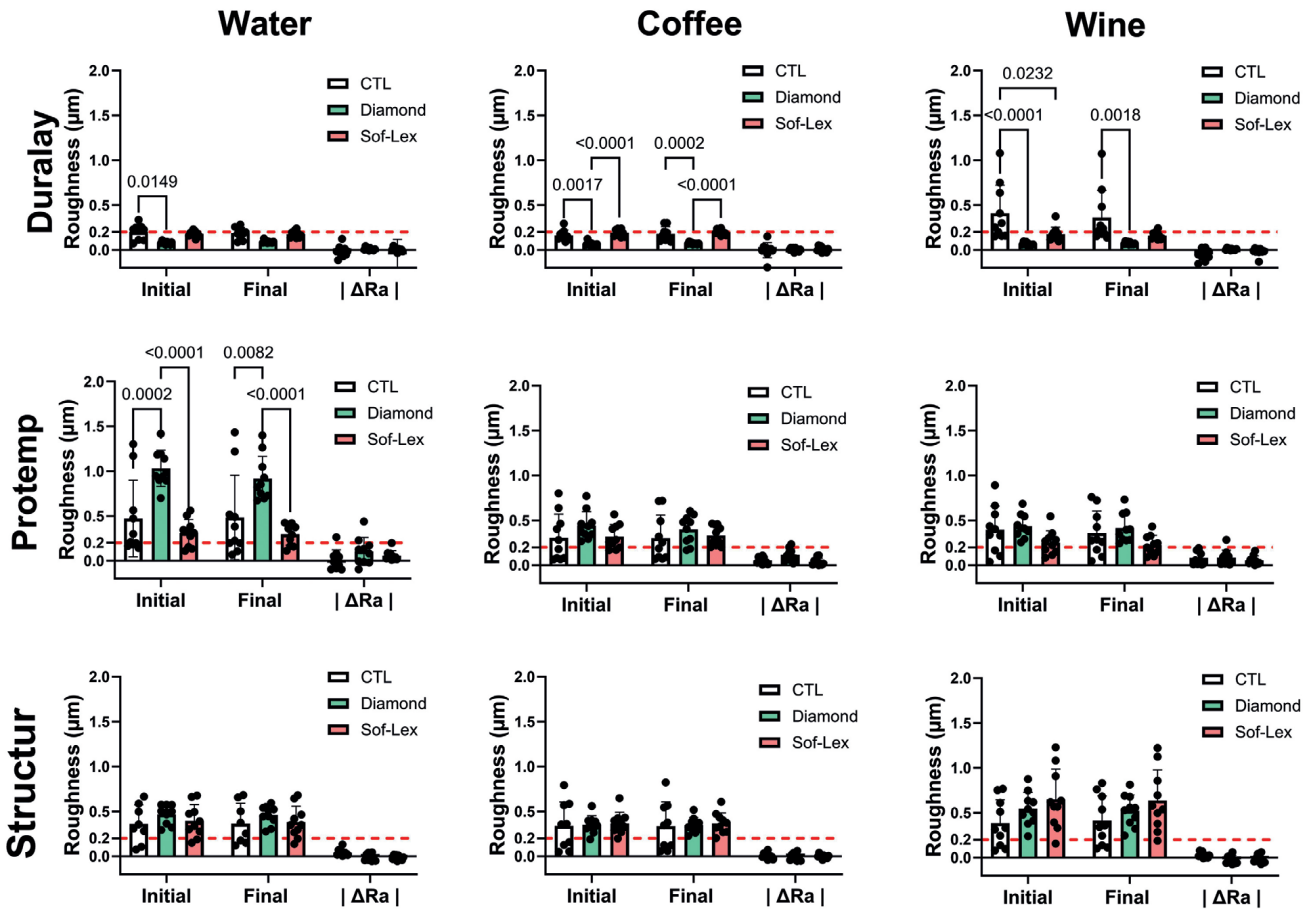
Mean and standard deviation in surface roughness (μm) of the materials. p-values are presented when a significant difference is observed between groups by one-way ANOVA followed by Tukey’s tests (*p*< 0.05, n = 10/group). The red dashed line indicates the clinically acceptable threshold.

Figure 4 shows that for all materials tested, significant color changes were noticeable after immersion in staining solutions for 30 days. The finishing and polishing systems were not able to affect the color stability of the chemically activated acrylic resin (*p* > 0.05). For the bis-acryl composite resins (Protemp and Structur 3), lower ∆E values were observed compared to control after immersion in staining solutions (*p* < 0.05).

## Discussion

The null hypotheses were rejected as the results showed that the finishing and polishing systems affected the surface roughness and color stability of the bis-acryl and chemically activated materials. Overall, a decrease in surface roughness and no changes in color stability were only observed for the acrylic resin. For bis-acryl composite resins, the finishing and polishing systems caused a significant decrease in ∆E values after immersion in staining solutions.

The surface roughness of restorative materials significantly impacts aesthetics, biofilm retention, and subsequently, periodontal health. Ideally, surface roughness values under 0.2 μm are considered clinically acceptable (16). Furthermore, a surface roughness of 0.3 μm can be detected by the tip of the tongue (17). Roughness values of provisional restoration materials vary widely in the literature, ranging from 0.14 to 1.98 μm for acrylic resins, and from 0.072 to 1.27 μm for bis-acryl resins (18). Accordingly, the surface values reported herein agree with the literature.

Surface roughness above 0.2 μm is a risk factor for biofilm accumulation and consequently caries and periodontal diseases (16,19). In this aspect, the effect of finishing and polishing systems on the surface of the resins used for temporization is clinically relevant. The results of this study showed that initial surface roughness was close to the clinically acceptable threshold. The 1-mm-thick glass slide positioned over the matrix during specimen preparation produces a specular surface with lower roughness values. This procedure forces the resinous monomers to the surface of the specimen providing smoothness, although water degradation cycles can alter this monomeric surface layer, causing high roughness values.

Another relevant aspect observed was that the finishing and polishing systems reduced the roughness of the acrylic resin, no effect, however, was observed for the bis-acryl composite resins. This is consistent with previous literature showing that acrylic resins polished with diamond paste produced smoother surfaces than those polished with aluminum oxide paste, and the average surface roughness of all bis-acryl composite materials was higher than those for methacrylate-based resins (20). In this case, the polishing systems might have removed the surface layer of monomers from the acrylic resin exposing the more resistant subsurface layer. After immersion in staining solutions, wine induced lower roughness in the acrylic resin, which was not observed for the bis-acryl groups. This might be related to pH-induced degradation of the organic matrix of the resin as wine may have some degradation effect on the composition of acrylic resins.

Chemically activated acrylic resins are based on the monomer methyl methacrylate while bis acryl composite resins present in their composition bifunctional acrylates that form cross-linking and combined with inorganic fillers. Regardless of the surface polishing technique used, the inherent chemistry of the material, resin matrix composition and the presence of inorganic particles, as well as their size and distribution, might have affected the results reported herein. As the manufacturers do not provide enough information on the composition and properties of these materials, an analysis of the components released from the materials would be valuable to better understand the results.

In this study, the color measurements were presented according to the Commission Internationale de l’éclairage L*, a*, and b* values. There’s a lack of agreement regarding the clinically acceptable range of color differences, and various studies in the literature have presented differing values for what is considered acceptable or noticeable ∆E values (1,12,21). According to (22), noticeable color differences are observed when ΔE values range from 3.5 to 5. Also, when these values are above 5, it gives the impression of two different colors.

The results showed that before immersion in the staining solutions, ∆E values were clinically accepTable (1,12,21) and would probably not have a noticeable effect. Immersion in coffee and red wine, however, significantly increased the ∆E values of the specimens, exceeding the threshold of perceptibility, aligning with findings from other studies (1,18,23,24). The results obtained herein showed that the finishing and polishing systems were able to significantly reduce the ∆E values of the bis-acryl resin materials when immersed in coffee or wine for 30 days. A more pronounced effect was observed for the Structur 4 polished with Diamond when compared with Sof-Lex discs.

The greater effect of the staining solutions on the bis-acryl composite resins can be attributed to the polarity of the material. Most bisacryl polymers are more polar than acrylic resins because of their high affinity to water and other polar liquids. Moreover, the larger inorganic filler particles present in the bis-acryl composite resins create surface irregularities, adding to color differences. Among the polishing systems used, aluminum oxide discs were more favorable to shade stability compared to rubber discs (Sof-lex®), even if the shade was modified after soaking in staining solutions.

Coffee induced greater shade change than red wine which agrees with the literature. The yellow dyes in coffee are less polar than the dyes present in wine. The absorption and penetration of dyes into the organic phase of the resin-based materials are probably due to the compatibility of the polymer phase with coffee-yellow dyes. Both coffee and wine contain a large amount of coloring agents, such as gallic acid, which could be another reason for the staining ability of these materials.

Overall, the combined results of surface roughness as well as color stability showed a better performance of aluminum oxide discs (diamond group) in chemically activated materials. While the findings presented here contribute significantly to our comprehension of finishing and polishing systems in acrylic and bis-acryl composite resins, it’s essential to approach them cautiously due to the inherent limitations of *in vitro* studies (1). Despite these constraints, *in vitro* research plays a pivotal role in unraveling the performance of dental materials. Factors such as degree of conversion, residual monomer percentages, porosity, mechanical and other optical properties, and material adaptation are imperative. Moreover, complementing these *in vitro* investigations, *in vivo* studies become crucial for assessing the mechanical and optical properties of these materials within the oral environment.

## Conclusions

The finishing and polishing systems influenced the surface roughness of the bis-acryl and chemically activated acrylic materials tested.

The chemically activated acrylic resin showed lower surface roughness and higher color stability when compared to the bis-acryl materials.

## Figures and Tables

**Table 1:** Materials tested and manufacturer’s information.

Material	Type	Composition	Shade	Mixing ratio	Manufacturer	Batch #
Duralay	Acrylic resin	Powder: polymethyl methacrylate (99.85%), diethyl phthalate (0.01% - 0.05%), benzoyl Peroxide (0.03% - 0.05%), and pigments (0.10%). Liquid: methyl methacrylate (99.92%), isopropyl alcohol (0.05%), and dimethyl p-toluidine (0.03%).	62	3:1	Reliance, São Paulo, Brazil	43926
Protemp 4	Bis acryl composite resins	Base: Bis-EMA and dieter-di methacrylate (50-60%), amorphous silica (20-30%), polyurethane methacrylate (10-20%), silane-treated silica (5- 10%). Catalyst: Ethanol (70-80%), silane-treated silica (<10%), and benzyl-phenyl-barbituric acid (<10%).	A2	10:1	3M ESPE, St. Paul Minnesota, USA	1200010525
Structur 3	Bis acryl composite resins	Bis-GMA, BHT, amines, benzoyl peroxide, dimethacrylates, glass Particles	A2	1:1	Voco, Cuxhaven, Germany	1200010525

**Table 2:** Finishing/polishing systems used in the study*.

Material	Composition	Mode of use	Manufacturer
Diamond Master	Diamond Pro Discs: polyester disc, adhesive, abrasive, silicone rubber. Granulation: thick, medium, and fine. - Diamond Flex felt disc: Polyester, adhesive, micro bristles, and silicone rubber. - Diamond Excel polishing paste: micronized diamond, lubricant base, thickener and emulsifier	Consistent circular motions, gradually reducing abrasiveness, punctuated by intermittent moistening to prevent overheating and subsequent surface alteration	FGM, Joinville, Santa Catarina, Brazil
Sof-Lex Discs	Set of three discs impregnated with Al2O3 particles: medium (40 μm), fine (24 μm), and ultra-fine (8 μm)	Intermittent and consistent motions within the gradual decrease of abrasiveness are alternated with periodic moistening to avoid excessive heat and subsequent surface damage.	3M ESPE, St. Paul Minnesota, USA

* Data provided by manufacturer datasheet

## Data Availability

The datasets used and/or analyzed during the current study are available from the corresponding author.

## References

[1] Morgado LB, Pedrosa MS, Medeiros IS (2024). Post-cure Heat Treatments Influence the Mechanical and Optical Properties of Acrylic and Bis-acryl Composite Resins.. Oper Dent.

[2] Burns DR, Beck DA, Nelson SK (2003). A review of selected dental literature on contemporary provisional fixed prosthodontic treatment: report of the Committee on Research in Fixed Prosthodontics of the Academy of Fixed Prosthodontics. J Prosthet Dent.

[3] Taylor M, Masood M, Mnatzaganian G (2021). Longevity of complete dentures: A systematic review and meta-analysis. J Prosthet Dent.

[4] Astudillo-Rubio D, Delgado-Gaete A, Bellot-Arcís C, Montiel-Company JM, Pascual-Moscardó A, Almerich-Silla JM (2018). Mechanical properties of provisional dental materials: A systematic review and meta-analysis. PLoS One.

[5] Raszewski Z, Nowakowska-Toporowska A, Nowakowska D, Więckiewicz W (2021). Update on acrylic resins used in dentistry. Mini Rev Med Chem.

[6] Köroğlu A, Sahin O, Dede DÖ, Yilmaz B (2016). Effect of different surface treatment methods on the surface roughness and color stability of interim prosthodontic materials. J Prosthet Dent.

[7] Rutkunas V, Sabaliauskas V, Mizutani H (2010). Effects of different food colorants and polishing techniques on color stability of provisional prosthetic materials. Dent Mater J.

[8] Magdy NM, Kola MZ, Alqahtani HH, Alqahtani MD, Alghmlas AS (2017). Evaluation of surface roughness of different direct resin-based composites. J Int Soc Prev Community Dent.

[9] Heintze SD, Forjanic M, Rousson V (2006). Surface roughness and gloss of dental materials as a function of force and polishing time in vitro. Dent Mater.

[10] Haselton DR, Diaz-Arnold AM, Dawson DV (2005). Color stability of provisional crown and fixed partial denture resins. J Prosthet Dent.

[11] Senawongse P, Pongprueksa P (2007). Surface roughness of nanofill and nanohybrid resin composites after polishing and brushing. J Esthet Restor Dent.

[12] Baldo V de O, Pedrosa M da S, Medeiros IS (2021). Post-cure heat treatments influence on mechanical and optical properties of resin composites. Braz Dent J.

[13] Vulović S, Stašić JN, Ilić J, Todorović M, Jevremović D, Milić-Lemić A (2023). Effect of different finishing and polishing procedures on surface roughness and microbial adhesion on highly-filled composites for injectable mold technique. J Esthet Restor Dent.

[14] Kobayashi M, Koi K, Wiskoski S, Watanabe H, Lewis S, Ferracane JL (2023). Isolated effect of filler particle size on surface properties of experimental resin composites before and after toothbrush abrasion. J Esthet Restor Dent.

[15] Paravina RD, Ghinea R, Herrera LJ, Bona AD, Igiel C, Linninger M (2015). Color difference thresholds in dentistry. J Esthet Restor Dent.

[16] Kim SK, Park JM, Lee MH, Jung JY, Li S, Wang X (2009). Effects of chairside polishing and brushing on surface roughness of acrylic denture base resins. J Wuhan Univ Technol Sci Ed.

[17] Jones CS, Billington RW, Pearson GJ (2004). The in vivo perception of roughness of restorations. Br Dent J.

[18] Soares IA, Leite PKB da S, Farias OR, Lemos GA, Batista AUD, Montenegro RV (2019). Polishing Methods' Influence on Color Stability and Roughness of 2 Provisional Prosthodontic Materials. J Prosthodont.

[19] Chung K hung (1994). Effects of finishing and polishing procedures on the surface texture of resin composites. Dent Mater.

[20] Şen D, Göller G, İşsever H (2002). The effect of two polishing pastes on the surface roughness of bis-acryl composite and methacrylate-based resins. J Prosthet Dent.

[21] Pedrosa M da S, Nogueira FN, Baldo V de O, Medeiros IS (2021). Changes in color and contrast ratio of resin composites after curing and storage in water. Saudi Dent J.

[22] Mokrzycki WS, Tatol M (2011). Colour difference δE - A survey. Mach Graph Vis.

[23] Silva J, Rafael CF, Vaz PCS, Fernandes JCAS, Volpato CAM (2019). Color stability of repairs on bis-acryl resin submitted to thermal aging and immersion in beverages. J Esthet Restor Dent.

[24] Barreto J de O, de Alencar-Silva FJ, Oliveira VC, Silva-Lovato CH, Silva PG, Regis RR (2019). The Effect of a Continuous Mechanical Polishing Protocol on Surface Roughness, Biofilm Adhesion, and Color Stability of Acrylic Resin Artificial Teeth. J Prosthodont.

